# Interpersonal Engagement Mediates the Relation between Maternal Affect and Externalising Behaviour in Young Children with Type 1 Diabetes

**DOI:** 10.1371/journal.pone.0097672

**Published:** 2014-06-06

**Authors:** Vivienne Chisholm, Andrea Gonzalez, Leslie Atkinson

**Affiliations:** 1 Division of Psychology & Sociology, Queen Margaret University, Edinburgh, Musselburgh, Scotland, United Kingdom; 2 Department of Psychiatry & Behavioural Neurosciences, McMaster University, Hamilton, Ontario, Canada; 3 Department of Psychology, Ryerson University, Toronto, Ontario, Canada; University of Western Brittany, France

## Abstract

Mother-child interactions around a shared activity have been shown to play a key role in the development of young children’s capacity to interact cooperatively with others. This evidence is particularly germane to type 1 diabetes (T1D) management in younger children where cooperation with parental treatment efforts is crucial for treatment success and where maternal distress and child behavioural problems are risk factors for treatment management, biomedical and psychological outcomes. In 49 4-to-8 year old children with T1D, we investigated whether the association between maternal affect and child problematic behaviour is mediated by mother-child interactions in the context of a T1D-relevant collaborative problem-solving activity. Mothers completed standardised measures of maternal and child psychological adjustment and interacted with their children in the problem-solving activity, analysed for quality of interpersonal engagement based on evaluations of maternal (sensitivity and cognitive stimulation) and dyadic (joint attention and warmth) behaviours. Mediation analyses confirmed the hypothesis that interpersonal engagement mediates the relation between maternal affective state and child behavioural problems. Specifically, more negative maternal affect is associated with lower levels of interpersonal engagement; these less engaged interactions in turn are associated with more behavioural problems in children. These findings are consistent with research involving typically developing children. The implications of our findings are twofold. First, in the context of psychological adjustment to T1D, maternal affect and mother-child interactions are 2 potential targets for interventions which promote cooperative interactions. Second, understanding and caring for children at biological risk requires attention to developmental psychology theory and method; in particular, research addressing parent-child cooperation carries both conceptual and clinical relevance.

## Introduction

A guiding principle in early childhood socialisation research is that young children’s capacity to interact cooperatively with others develops through social experience. Fundamental to this research is the premise that in early childhood, children’s ability to cooperate with environmental demands and expectations in the short-term and, to self-regulate in accordance with such external exigencies in the long-term, is developed through participation in shared activities with adults such as parents [1]. This view has particular resonance in relation to small children with type 1 diabetes (T1D) where treatment management must follow a developmental trajectory from cooperation with parental treatment efforts in early childhood to independent self-care capability in adolescence. T1D diagnosis heralds a lifelong commitment to a complex regimen [2], based on diet and insulin therapy, and designed to approximate normal blood glucose (BG) levels. Although parents of young children have complete responsibility for treatment implementation, children’s cooperation with parental efforts is essential for treatment success. T1D research with younger children indicates that maternal distress, parent-child interaction difficulties and child behavioural problems are risk factors for poorer treatment management and more adverse biomedical and psychological outcomes [3]–[9]. However, studies have not explored these risk factors as they occur simultaneously in younger children, nor have they addressed the mechanism whereby they are linked. We investigated whether the association between maternal affect and problematic behaviour in young children with T1D is mediated by mother-child interaction.

Our proposed model, examining the role of maternal affective state, mother-child interaction and child behaviour difficulties in T1D in early childhood is important for three reasons. First, the incidence of T1D is increasing dramatically in young children worldwide, an ‘accelerating epidemic’ [10], [11], carrying significant health and resource implications [12]. Second, younger age at T1D onset increases the risk of chronic microvascular and macrovascular complications [10]. Thus good early adjustment is crucial because management patterns tend to be established early in disease onset [13] and young people with psychological and treatment compliance problems have much poorer prognoses [14], including greater risk for premature death [15]. However, younger children have been relatively overlooked in the research literature although preventive interventions may be most effective when delivered in early childhood [16]. Third, and of particular relevance here, the mother-child relation is the primary social arena in which daily treatment takes place [17].

### Role of Maternal Affect

The caretaking responsibility for parents of young children with T1D is enormous, with vigilant monitoring of children’s well-being and treatment decision making (e.g., in response to BG fluctuations) forming part of daily life [18]. In healthy populations, behavioural problems are common in preschool- and primary school-aged children [19] and correlate with negative maternal affect [20]. In comparison to healthy children, parents of young children with T1D report more difficulty with misbehaviour. Behavioural difficulties include potentially health-compromising, but developmentally typical, problems like non-cooperative behaviour at mealtimes in addition to general behavioural problems [4], [7], [8], [18], [21], [22]. Moreover, misbehaviour of this kind may hamper parental treatment efforts and amplify parental stress [4]. In addition, mothers who report more frequent misbehaviour also report spending more time in diabetes management and believe that diabetes has a greater impact on their disciplinary practices, including engaging in over-reactive discipline [8]. Indeed**,** Patton et al. [7] found that parents’ use of ineffective or coercive strategies such as commands or physical prompts during family mealtimes correlated with poorer BG control, poorer dietary adherence and disruptive behaviours such as spitting out food or leaving the table during mealtimes. Consistent with these findings, in a population-based study of 4-year-old children, mothers of children with chronic illness reported more disruptive behaviours (e.g., quarrelling, temper tantrums) than mothers of healthy children [23]. In a sample of school-aged children followed longitudinally, both younger age at diagnosis and externalising behaviours predicted multiple hospitalisations for complications caused by poor BG control [5]. Mothers of young children with T1D are at increased risk for greater emotional distress [4], [17] and are more likely to experience psychological difficulty than other family members such as fathers or nondiabetic siblings [9], [24]. In sum, for both mother and child, the impact of T1D management is considerable and for parents, the challenge is to ensure children’s treatment cooperation with minimal negative psychological consequences.

### Importance of Mother-child Interactions

We know from developmental research on typical populations that in early childhood, quality of mother-child interaction during shared activity predicts children’s behavioural adjustment both concurrently and prospectively [25], [26]. This has been demonstrated across a range of contexts pertaining to both problem-solving and free play activities [26], [27]. Moreover, children who participate in lower levels of shared activity with mothers have more behaviour problems [28]. Further, research shows consistent associations between specific features of maternal behaviour and dyadic interactions during shared activity and child behavioural adjustment. With respect to maternal behaviours, behaviours which are sensitive (i.e., attuned to the child’s signals) and stimulating (i.e., promote learning and understanding) predict fewer behavioural problems [25],[29]–[31]. Regarding dyadic interactions, interactions characterized by higher levels of joint attention to a shared activity and expressions of warmth and affection also predict fewer behavioural problems [29], [32]. In addition, symptoms of maternal negative affect, at both clinical and sub-clinical levels, predict poorer quality parenting and poorer quality parent-child interactions along the dimensions considered here (i.e., maternal sensitivity, cognitive stimulation, joint attention and warmth) as well as child behavioural problems [19], [33]–[36], with mediation analyses indicating that such features of parent-child interactions provide the path through which maternal affective state influences child behavioural adjustment [37], [38]. Thus, in developmental research, there is increasing evidence that early childhood externalising problems, in particular, are influenced by features of parent-child interactions such as the absence of parental positivity and low levels of dyadic mutuality [32], [38]. These findings carry fundamental implications for the study of T1D adjustment in younger children because treatment management is an inherently collaborative activity, based on an array of daily self-care behaviours (e.g., BG testing, eating a carbohydrate-regulated diet, insulin administration) which require not only a general understanding of diabetes, but also the ability to skilfully apply this knowledge in daily problem-solving situations. Moreover, as indicated, children’s cooperation is essential for treatment success, particularly in areas like dietary management, the treatment component most strongly associated with mother-child interaction difficulties and child behavioural problems [3], [39].

Observational studies of mother-young child interactions in a T1D-specific collaborative problem-solving activity, although scant, demonstrate associations between specific maternal behaviours and differential child adjustment outcomes. Specifically, maternal utterances promoting child participation in the activity behaviourally (e.g., through suggestions), and cognitively (e.g., through questions), correlate with better treatment adherence, better BG control and better child psychological adjustment [39]. In contrast, negative communications like ambiguous messages (e.g., criticism paired with a smile, sarcasm) correlate with both child adjustment problems and poorer treatment adherence [40]. Collectively, these findings highlight the influence of quality of mother-child interactions on adjustment in young children with TD but they do not provide insight into the influence of maternal affect. In this study, we investigate the mechanism by which maternal affective state influences child behavioural adjustment outcomes in young children with T1D. Extrapolating from findings in the developmental research literature discussed above, we hypothesised that quality of mother-child interaction mediates the relation between maternal affective state and child behavioural problems.

## Materials and Methods

### Participants

Study participants were 49 children (30 boys) and their mothers participating in a T1D home management study in Scotland for younger children. We approached all families with children with T1D aged 8 years and younger registered at the Diabetes Clinic. This entailed 94 invitations. Of this group, 65 families (69%) consented to participation. The 49 children included here are the older children in this sample. We excluded children under 48 months because the problem-solving activity entails the classification of food items based on food groups in the context of a birthday party meal (see below, Mother-child food selection problem-solving activity).

Children’s mean age was 82.08 months (standard deviation (SD), 17.41); mean age at diagnosis, 61.75 months (SD, 26.00). Children’s diabetic control was assessed through measurement of glycosylated haemoglobin levels whereby percentage of haemoglobin with glucose attached is assessed, with a higher value indicating poorer BG control. Mean HBA1c level was 7.99% (SD, 1.19%). Parental occupation was classified according to the National Statistics Socio-Economic Classification (NS–SEC) class designations [41]: 47.73% were in social classes 1 and 2, the higher managerial and professional classes; 29.55% were in social classes 3, 4 and 5 (e.g., small employers, account workers, lower supervisory positions); 22.72% were in social classes 6, 7 and 8 (routine and semi-routine jobs and unemployed); 82.46% of the mothers were in stable relationships (either married or common-law) such that their children lived in dual-parent households.

### Ethics Statement

The National Health Service (NHS) Lothian Health Board, Paediatric and Reproductive Medicine Sub-Committee gave ethical approval to this study. Approval was given to all the materials and procedures described here (see below) as well as an Information Sheet and a Patient Consent. Form (a standardised form issued by Lothian Health Board). The ethical approval process took place before we commenced participant recruitment and data collection. With respect to participant recruitment, mothers of young children were given the Information Sheet and the Patient Consent Form. The Information Sheet contained an invitation to mothers and their children to participate in a T1D home management study and a description of the study. It advised mothers of their right to either decline participation or to withdraw from the study (after provision of consent) without impact on the services they were receiving from the hospital. Mothers were also requested (in the Information Sheet) to discuss the study with their children prior to providing consent. In addition, mothers were asked to confirm willingness to participate by signing the Consent Form itself and returning the form to the investigators. A copy of the signed Patient Consent Form was sent to. mothers for their own records.

### Measures

Mothers completed standardised measures of their own affective state and child psychological adjustment. Mothers and children were observed at home engaging in a 20-minute, videotaped problem-solving activity.

#### The bipolar profile of mood states (POMS-BI) [42]

The POMS-BI Contains Six 12-Item Subscales (Composed/Anxious, Agreeable/Hostile, Elated/Depressed, Confident/Unsure, Energetic/Tired and Clearheaded/Confused); Respondents Are Requested to Rate, on a 4-Point Scale, Their Feelings ‘during the past Week Including Today’. This Instrument Was Selected Because It Measures Both Negative and Positive Affect and Is Intended for Use with Both Clinical and Nonclinical Populations [42], [43]. We Based Analyses on the Total Positive Affect Score Derived from the 6 Subscale T Scores, in Accordance with the Manual [42] and Previously Published Studies [44]. Each Subscale Has a Mean of 50 and a Standard Deviation (SD) of 10 [42]. a Higher Score Indicates a More Positive Emotional State. This Instrument Shows Good Internal Consistency and Test-Retest Reliability across the Subscales [42], [43], [45]. the Validity of This Instrument Is Also Well-Established across a Range of Contexts [46]–[48], Including Maternal Cognitions regarding Interactions with Young Children in Stressful Situations Such as Mealtimes [49], and Parental Distress in Relation to Decision-Making for Children with Life Threatening Illnesses [50].

#### The child behavior checklist – parent report (CBCL-P/4-18) [51]

The CBCL- P measures child Internalising (emotional), Externalising (behavioural), and Total problems, with higher T scores indicating poorer psychological adjustment. This instrument is widely used in child health and early childhood compliance research. It has good internal consistency, test-retest reliability and validity in typically developing populations [51]. In an evidence-based assessment of the reliability and validity of measures assessing psychological adjustment in paediatric populations, the CBCL met the empirical criteria for ‘well-established’ [52].

Analyses here are based on Externalising problems scores because behavioural problems are common in young paediatric populations with T1D, posing distinctive caretaking challenges for mothers [4], [8], [16] and predicting poorer health outcomes [5], [53]. In older children and adolescents with T1D, externalising behaviour problems are associated with poorer parental relationship quality, treatment nonadherence, and poorer glycaemic control [54]–[58]. In typically developing populations, early childhood externalising problems in particular are associated with compliance problems, less parental positivity, less mutuality, and more disruptive interactions in mother-child problem-solving contexts and predict poorer mental health and developmental outcomes [28]–[30], [32], [38].

### Mother-child Food Selection Problem-solving Activity

We designed a board game whereby children select food for their birthday party [39], [40]. The main food categories (Bread, cereals, rice, pasta; Sweets, oils, fats; Meat, fish, poultry, beans, nuts; Fruit; Vegetables; Cheese, milk, yoghurts) are displayed in bright colours on a laminated board, with laminated cardboard replicas of individual food items (e.g., an apple) attached by velcro to their respective categories (e.g., Fruit) on the board. The Birthday Game comprises two components: 1) Children select, from the ‘Shopping Platter’, food items and put them in their shopping basket. 2) Children place the items they have in their shopping basket on the ‘Birthday Platter’. Here the child must decide the placement of the items according to food category (e.g., apples go in Fruits, birthday cake in Sweets, Oils & Fats, etc.). Prior to playing, mothers and children are instructed verbally how to play the game and are asked to take into account the child’s diabetes when planning the birthday party meal. Mothers are also provided with a written copy of instructions.

We used a ‘birthday party’ as the problem-solving context because it requires mothers and children to plan a meal that accommodates the child’s dietary requirements in the context of a peer-related event that is common to young children’s social lives. In contrast to studies involving young children with T1D based on observations of family mealtimes [7], [21], the context here differs in 3 respects: 1) It provides a standardised format for the observation of mother-child collaborative interactions across study participants. 2) It permits focus on the interaction of mother and child in particular. This is especially important because the mother-child relation is the primary social arena in which daily treatment takes place [17] and mothers and children with T1D are at greater risk for psychological and relationship difficulty compared to other family members [9]. 3) Mothers and children are presented with the task of planning a meal from a wide array of choices. This approach is conceptually and clinically meaningful in the context of T1D because problem-solving skills such as planning and reasoning are essential for effective T1D management [59]. Research in developmental psychology shows that social experiences with parents in collaborative problem-solving activities are crucial in early childhood for the development of autonomous problem-solving skills as well as the capacity to interact cooperatively with others which in turn are influenced by non-cognitive factors such as maternal affect, child externalising behaviours and interaction quality [29], [32], [34], [35]. In sum, this activity provided a standardised paradigm by which we could observe (*in vivo*, in an emotionally potent and T1D-relevant activity) key features of mother and child interpersonal engagement such as mutual affection and maternal sensitivity (see *Videotape Analysis* below). This activity has been validated in previous research where communicative differences between mother-child dyads (e.g., in control style or communication congruence) discriminated differential psychological, adherence and BG control outcomes in children [39], [40].

Developmental research shows that children in the entire age range considered here have cognitive understandings in the food domain which enable participation in this activity. For example, they can classify food items into script (i.e., situations when foods are served such as breakfast or birthday party), taxonomic (e.g., fruits) and evaluative (e.g., ‘unhealthy’ or ‘healthy’ foods) categories [60], [61] and further, are able to cross-classify single food items into taxonomic and script categories (e.g., ice cream is a dairy product and a birthday party food; milk is a diary product and a snack) and can use these categories to make inductive inferences about foods [61]–[63]. Furthermore, developmental research shows that children in the age range considered here can participate in joint conversations with mothers about past experiences and activities [64] and view mothers as an important source of information about food [65], important considerations in light of the interactive nature of this task. In sum, collectively, these findings indicate that the problem-solving activity used here is developmentally appropriate and meaningful for children in the age range in this sample.

### Videotape Analysis

The Birthday Game observational data were analysed using the qualitative rating scales of maternal sensitivity, maternal stimulation and dyadic interaction developed by the National Institute of Child Health and Human Development Early Child Care Research Network (NICHD ECCRN) [25]. We used these scales for 3 reasons: 1) they were developed to study young children and parents engaging in collaborative problem-solving and have established validity and reliability. For example, these scales discriminate features of mother-child interactions (e.g., maternal sensitivity, joint attention to task, affective mutuality) which predict externalising behaviours in early childhood [25] as well as outcomes not measured here, such as language and academic outcomes [31]. 2) They allow qualitative analysis of maternal and dyadic verbal and nonverbal communication, and 3) They allow evaluation of maternal emotional and instrumental support for children’s activities.

For all categories, we used a 5-point rating scale, providing criteria for each point to facilitate coding. Each rating for each category is based on an overall evaluation of the entire session for each mother-child dyad. An analytic approach based on rating scales is an empirically attractive complement to maternal report measures because they provide a more objective perspective on interactions and explain variance in subsequent child outcomes beyond the variance predicted by maternal or interviewer report [26].

### Observational Categories

Following the analytic technique developed for the NICHD ECCRN study [25], maternal sensitivity and adult stimulation composite scores were derived by summing each of the respective sub-categories indicated below:

### Maternal Sensitivity

#### Supportive presence

Extent of maternal level of positive regard and emotional support for the child, e.g., smiling at and praising the child, responsive to the child’s behaviour vs. being aloof and emotionally unavailable.

#### Respect for autonomy

Extent to which mother behaves in a manner that acknowledges the child’s individuality and validity of his/her actions, e.g., giving decision making responsibility to the child, “You can decide what you’re gonna have” vs. interfering with the child’s choices, “Wait a minute, you wouldn’t have lemon at your party”.

#### Hostility

Extent to which mothers express anger towards or rejection of the child or his/her behaviours. A parent who receives a high score on this scale would make overt expressions of criticism, e.g., mother takes item from child’s hand, saying “No! You don’t even know what that is”, “Don’t be silly”. A parent scoring low on this scale would rarely direct hostility to the child.

Hostility was reverse scored in the calculation of the composite variable, Maternal Sensitivity.

### Maternal Stimulation

#### Stimulation of cognitive development

Extent to which mother promotes the child’s understanding of the activity and T1D treatment principles, e.g., “What would you do, because you’d be running about, and you’d need lots of energy, so what you’d be needing, you know, Mum’s always telling you about carbohydrates and that’s things like… ?” vs. “No, you know we don’t eat chocolate”.

#### Quality of assistance

Extent to which mother structures the situation in the context of task objectives and provides hints and corrections, e.g., the child is putting the gingerbread man in the Vegetables section on the ‘Birthday Platter’. Mother puts her hand over child’s hand and says “No, ‘cause you know where the gingerbread man goes? … Do you think he’s got a lot of sugar in him?”; “If you put them in your basket, then what we do is we put them on the plate underneath once we’ve done our shopping.” vs. “That would go there”.

### Dyadic Interaction

In addition, we formed a composite Dyadic Interaction score by summing the two sub-categories below.

#### Goal-directed partnership

Extent to which parent and child work together, both contributing to the activity, e.g., “We’ll have a wee look and then we’ll decide” vs. mother passively watching her child while s/he selects items for the party. Here, parent and child show no shared involvement in the activity either verbally (e.g., by discussing food choices) or nonverbally (e.g., by pointing food items out to each other or by mother turning the board around for the child while s/he puts items in the basket).

#### Affective mutuality

Extent to which mother and child convey an impression of warmth and intimacy, e.g., expressions of affection such as kissing or leaning in towards each other such that they are in physical contact or terms of endearment or fun, e.g., “Whoops, you’re losing your bananas, honey”, “My head is feeling like I would like some ice cream” vs. leaning away from each other and not expressing affection neither verbally or nonverbally.

### Observational Data Coding Procedure

Data were coded by 2 observers, both with honours level psychology undergraduate degrees. They were blind to all other information about the families. To ascertain inter-observer agreement, the observers independently rated all interaction tapes. Intra-class correlations across maternal sensitivity, adult stimulation, and dyadic interaction varied between.81 and.82 (p<.0005). Mean ratings were used for the purpose of data analysis.

### Data Analysis

We assessed bivariate relations amongst study variables using Pearson product-moment, point-biserial, and phi correlations, as appropriate. We assessed the indirect effect of maternal affect on child behaviour problems, via mother-child interaction, using ordinary least squares regression with bootstrapping, 5000 resamples, 95% bias corrected and accelerated confidence intervals, as described by Hayes [66], [67].

## Results

### Descriptive Statistics and Study Variables

Mean externalising score was 47.78 (SD, 9.91); this score is within normal range functioning and comparable to mean externalising scores in early childhood compliance and self-regulation research with nonpaediatric [29], [30], [36] and paediatric populations with T1D [53]. Four children (8% of sample) attained scores in the clinical range of functioning (≥64).

Mean total POMS-BI T score was 294.98 (SD, 39.58). Mean sub-scale T scores ranged from 44.1 (SD, 8.73) for agreeable/hostile to 52.2 (SD, 8.97) for clearheaded/confused which are within normal range functioning, specifically 40≤T≤60. These findings are comparable to mean sub-scale T scores obtained in a nonpaediatric [49] and paediatric [50] populations.

We assessed relations amongst the Birthday Game observational variables, finding Pearson product-moment correlations of.53 (sensitivity with adult stimulation), .77 (sensitivity with dyadic interaction), and.68 (stimulation with dyadic interaction) (*p*<.0005 in every case). These correlations confirmed our decision to form a composite variable, termed ‘Interpersonal Engagement’, derived by summing the observational categories (Maternal sensitivity, Maternal stimulation, Dyadic interaction). Other considerations also informed this decision. Specifically, the development of a single variable attenuated the risk of type I error and the developmental research literature shows that the behaviours included in interpersonal engagement are associated with maternal affective state and behavioural adjustment in young children [32], [34], [35], [38].

### Assessing for Confounds

Child sex is related to maternal affect such that mothers with female children report more positive mood than mothers of male children. No other significant relations emerged between background demographic and medical variables (sex, age, age at diagnosis, T1D duration, social class, and HbA1c), on the one hand, and maternal affect, mother-child interpersonal engagement, and child externalising problems, on the other (Table 1). Because none of the background variables were related to the outcome variable, externalising problems, they were not included in further analyses as potential confounds.

**Table 1:** Intercorrelations of all variables used in this study.

	Sex	Age	Age atDiagnosis	T1D Duration	SES	HbA1clevel	CBCL Externalizing	MaternalPOMS-BI
Age	−.059							
Age atdiagnosis	.025	.514*						
T1DDuration	−.025	.180	−.744*					
SES	−.068	.010	.082	−.112				
HbA1c level	.093	.116	−.114	.204	−.163			
CBCL externalizing	.132	−.176	−.013	−.120	.274	−.116		
Maternal POMS-BI	−.289*	.140	.043	−.264	−.234	−.036	−.208	
Interpersonal Engagement	−.081	.068	.232	−.194	−.072	−.111	−.372*	.323*

Note: SES = Socio-economic status, as assessed with the National Statistics Socio-Economic Classification [41]; HbA1c = haemoglobin A1c; CBCL = Child Behavior Checklist [51]; POMS-BI = Bipolar Profile of Mood States [42].

*p<.01.

### Bivariate Associations among Target Study Variables

All associations amongst target variables are shown in Table 1. More positive maternal affect correlated significantly with more interpersonal engagement during the problem-solving activity. More interpersonal engagement correlated significantly with fewer externalising problems. The correlation between maternal affect and externalising problems approached significance, with more positive affect correlating with fewer externalising problems (*p*<.10).

### Testing the Indirect Effect of Maternal Affect on Externalising Problems via Interpersonal Engagement

**Figure 1:**
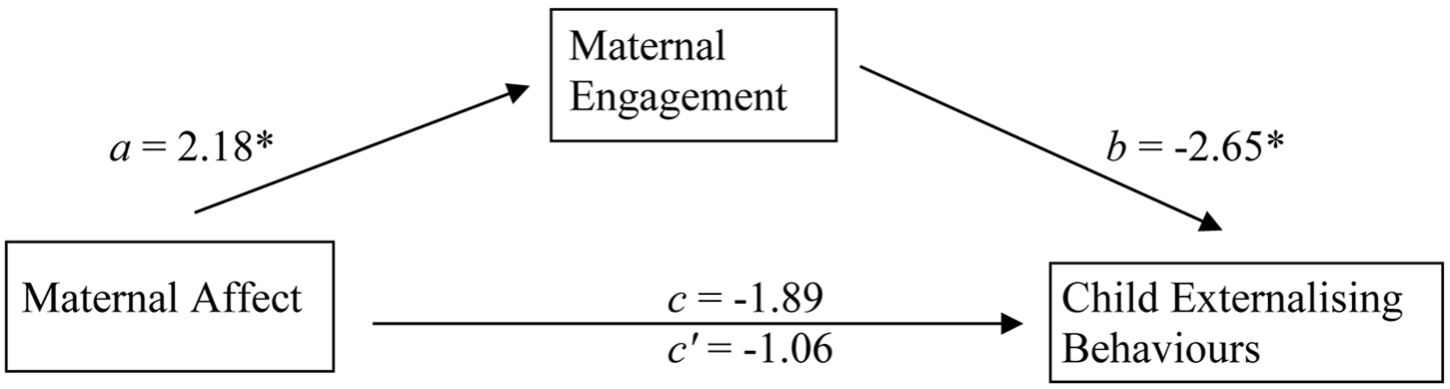
Indirect impact of maternal affect on child externalizing behaviours via maternal engagement. Note: *a*, *b*  =  direct effects; *c*  =  total (direct + indirect) effect; *c’*  =  direct (total– direct) effect. All coefficients are standardised. The difference between c and c’ is significant (95% bias corrected and accelerated confidence interval  = −.081 to −.002). The entire model accounts for 16% of the variance (adjusted *R* ^2^ = 12.14). *p<.05.

We assessed the hypothesis that maternal affect exerts an indirect effect on child externalising behaviours via interpersonal engagement, as depicted in Figure 1. As mentioned, we used ordinary least squares regression with bootstrapping [66], [67]. The findings represent the means of the bootstrap distributions. Essentially, this procedure assesses the hypothesis that the association between the dependent and independent variables (child externalising behaviour and maternal affect, respectively) is significantly attenuated by the addition of the mediator (interpersonal engagement) into the equation. 1) Assessing the relation between maternal affect and interpersonal engagement, *a* = .045, standard error (*SE*)  = .017, *t* = 2.55, *p*<.05. (2) Assessing the direct effect of interpersonal engagement on child externalising behaviour, *b* = −.620, *SE* = .284, *t* = 2.19, *p*<.05. (3) Assessing the total effect of maternal affect on child externalising behaviour, *c* = −.066, *SE* = .035, *t* = 1.88, *p*<.07. It should be noted in this regard that although early work on mediation specified a significant relation between dependent and independent variables as a criterion for mediation [68], this relation is no longer considered necessary [69]. (4) Assessing the direct effect of maternal affect on child externalising behaviour (i.e., the effect of maternal affect on child externalising behaviour, independent of interpersonal engagement), *c’* = −.038, *SE* = .036, *t* = 1.06, *p* = .29; i.e., importantly, when interpersonal engagement is entered into the equation, the association between maternal affect and child externalising behaviour diminishes significantly (95% bias corrected and accelerated confidence interval  = −.081 to −.002). The overall model, regressing child externalising behaviour on maternal affect and interpersonal engagement, proved significant, *F*(2, 46)  = 4.29, *p*<.05, accounting for 16% of the variance (adjusted *R* ^2^ = .121). These analyses are consistent with the hypothesized model suggesting that maternal affect has an indirect influence on child externalising behaviours via interpersonal engagement. For the sake of clarity, these results are shown in Figure 1.

## Discussion

Although younger children with T1D are relatively understudied in the illness adjustment literature, research findings consistently indicate that maternal distress, parent-child interaction difficulties and child behavioural problems are potent risk factors for more adverse outcomes [3]–[9]. The purpose of this study was to investigate a mediation model, based on this triad of risk factors, in which we proposed that mother-child interactions provide the conduit through which maternal affect influences behavioural adjustment in young children with T1D. In the context of a collaborative problem-solving activity, we found that specific indices of interpersonal engagement comprising maternal (i.e., sensitivity and cognitive stimulation) and dyadic (i.e., joint attention and warmth) behaviours, which intercorrelate between *r* = .53 and.77, appear to mediate the relation between maternal affective state and child behavioural problems. While we adopted a cross-sectional, correlation-based approach to mediation, which precludes causal certainly, our findings are consistent with a theoretical model suggesting that maternal affect influences quality of interpersonal engagement which influences level of child behavioural problems. To our knowledge, this is the first study to empirically demonstrate a potential mediating mechanism between maternal affect and externalising problems in young children with T1D.

Our findings are consistent with the developmental literature in indicating that more negative maternal affect is associated with lower levels of maternal sensitivity and cognitive stimulation, in addition to less joint attention to the problem-solving activity and less mutual warmth. These less engaged interactions in turn lead to more externalising problems in children [28], [32], [34]–[36], [38]. For example, Goldsmith and Rogoff found that nondysphoric mothers were more sensitive than dysphoric mothers to children’s level of understanding and were more likely to share decision-making during food and picture classification tasks [34]. Foster, Garber and Durlak found that maternal ‘positivity’ (e.g., praise, warmth, assistance) during maze and word game puzzles partially mediated the relation between symptoms of maternal depression and child externalising symptoms [38]. A longitudinal study, following youngsters from infancy to adolescence, found that adolescents exposed to chronic symptoms of maternal dysphoria from early childhood, even at subclinical levels, reported more externalising problems and more risky behaviours [33]. Thus, in the case of young children with T1D, externalising problems may not only make daily disease management more difficult [4], they may also be harbingers of future difficulty. For example, findings from cross-sectional and longitudinal research involving adolescents with T1D show that externalising behaviour problems are associated with poorer glycemic control, poorer adherence, and poorer parental relationship quality [54]–[57]. With respect to mental health, Northam, Mattthews, Anderson, Cameron and Werther found that parent-reported externalising problems at T1D diagnosis in childhood predicted both affective and behavioural mental health problems 10 years later in adolescence, suggesting that childhood behavioural problems may be the developmental precursor of a range of psychopathologies [6]. The importance of preventing the development of such adverse trajectories is amplified by evidence that long-term microvascular complications may have their origins in poor diabetic control in adolescence when psychological and behavioural problems often interfere with treatment adherence [70].

Our study limitations are as follows. First, there is controversy regarding the use of cross-sectional data based on concurrent associations. While cross-sectional designs are typical in mediation research and it has been argued “strongly” that such data are appropriate ([71], p. 89), it has also been argued that such data may predispose towards bias, either inflating or deflating the estimates of longitudinal direct and indirect effects [72], [73]. The cross-sectional nature of the current design, and its correlational nature, also may confound the direction of effect; for example, it may be that externalising child behaviours elicit more negative maternal behaviour or that externalising child behaviour contributes to maternal negative affect consistent with transactional conceptualisations of the development of children’s behaviour problems [19]. In addition, the CBCL pertains to child behaviour over the past six months, while the POMS-BI assesses behaviour over the past week, further confounding causal inferences. While there are strong theoretical reasons to support the model we propose [68], and solid theory is ample justification for using cross-sectional data in mediation modeling [71], nevertheless, we do recommend the test of alternative models using longitudinal designs. Longitudinal research is also necessary to understand the influence of maternal affect and mother-child relations in early childhood on differential T1D adjustment trajectories, particularly as children grow older, form relationships outside the family, and assume greater responsibility for their care. Second, we did not assess the contribution of fathers to T1D adjustment quality. The developmental and clinical psychology research literatures demonstrate that fathers affect both developmental and mental health outcomes in their children directly (e.g., through the quality of interactions with children [32]) and indirectly (e.g., through the quality of relationship with mothers [20]). On the other hand, the illness adjustment literature indicates that mothers tend to be children’s primary caretakers and are at greater risk for distress compared to other family members [9], [17] suggesting that mothers in particular should be the focus of intervention strategies. Third, our sample size is small, predominantly middle class and comprising dual-parent households, of European descent, and drawn from a single site; these factors potentially constrain the generalisability of our findings. In addition, we acknowledge that the maternal report of child externalising behaviour may be prone to bias (e.g., mothers with low mood might over-report child difficulties). Although the CBCL has been strongly validated [51], [52], replication of the mediation model shown here using alternate methodologies (e.g., observation of child behaviour) would be useful.

However, our findings are consistent with both developmental psychology and T1D paediatric research involving racially-mixed and economically deprived populations in demonstrating that specific features of parent-child interactions such as positive affect, warmth, sensitivity and joint focus foster more favourable outcomes [26], [32], [55], [58]. To illustrate, Deater-Deckard, Atzaba-Poria and Pike [32] found that greater dyadic mutuality and positive affect in in young typically developing children and their parents predicted fewer externalising behaviours in children across gender, ethnic and socioeconomic groups. Other studies involving young children indicate that SES increases the risk externalising behaviours through its impact on parenting behaviours such as neglect and intrusiveness [74]. Consistent with these findings, and with regard to T1D, in racially mixed and economically deprived populations, child externalising behaviours and features of parent-child relations such as low cohesion or critical parenting are associated with poorer diabetic outcomes [55], [58]. Also, this was a well-adjusted sample; only 8% evinced clinically significant levels of difficulty. Psychological adjustment difficulties and treatment adherence problems tend to increase in late childhood and adolescence when children have greater self-care responsibility [15], [75], underscoring the importance of early childhood preventive interventions [16]. Nevertheless, these considerations notwithstanding, this is the first study we are aware of showing the mediated path by which maternal affect in the context of a collaborative activity may influence child outcome among young children with T1D. The model may serve as a basic platform upon which to expand our understanding of mechanism; the addition of further independent variables, mediators, and moderators would augment our understanding of developmental processes linking maternal factors and child behaviour in the context of T1D.

Most interventions are developed for adolescents when risk for treatment nonadherence is highest. By contrast, little attention is given to preventive interventions in early childhood which could attenuate the risk of adverse trajectories [16]. This is a significant oversight because T1D management patterns tend to be established early in disease onset [13]. Our findings highlight the contribution that developmental psychology theory and method can make to the study and care of young children at biological risk in providing insight into core features of interpersonal engagement in the mother-child relation that influence child adjustment. The findings suggest the importance of two potential targets of intervention, maternal affective state and mother-child interaction. In this regard, for example, Huebner [76] showed that a short-term educational intervention decreased parent-reported stress and improved observed parent-child interaction. This finding was demonstrated across varied populations. The programme itself taught parents how to identify circumstances that strained parent-child interactions and provided anticipatory guidance, support, and skills training to the parents. Or again, Moss, Dubois-Comtois, Cyr, Tarabulsy, St-Laurent, and Bernier [77] demonstrated that a brief, attachment-based intervention focused on the parent–child dyad and improved parental sensitivity effectively reduced child externalising behaviour. The intervention included discussion of attachment/emotion regulation themes and video feedback of parent–child interactions. Such interventions applied in the T1D context could promote positive maternal affect and equip mothers with the parenting behaviours they need to promote cooperative interactions with their child around T1D-related tasks. Early interventions of this kind may be the first step in establishing optimal treatment management trajectories in young children and averting trajectories which lead to adverse outcomes.
